# Tunnel Josephson Junction with Spin–Orbit/Ferromagnetic Valve

**DOI:** 10.3390/nano13131970

**Published:** 2023-06-28

**Authors:** Alexey Neilo, Sergey Bakurskiy, Nikolay Klenov, Igor Soloviev, Mikhail Kupriyanov

**Affiliations:** 1 National University of Science and Technology MISIS, 119049 Moscow, Russia; aleks.neilo@yandex.ru (A.N.); igor.soloviev@gmail.com (I.S.); mkupr@pn.sinp.msu.ru (M.K.); 2 Skobeltsyn Institute of Nuclear Physics, Lomonosov Moscow State University, 119991 Moscow, Russia; 3 Faculty of Physics, Moscow State University, 119991 Moscow, Russia

**Keywords:** Josephson junction, spin–orbit interaction, ferromagnetic, spin valve, spintronics, superconducting quantum computers

## Abstract

We have theoretically studied the transport properties of the SIsN${}_{SO}$F structure consisting of thick (S) and thin (s) films of superconductor, an insulator layer (I), a thin film of normal metal with spin–orbit interaction (SOI) (N${}_{SO}$), and a monodomain ferromagnetic layer (F). The interplay between superconductivity, ferromagnetism, and spin–orbit interaction allows the critical current of this Josephson junction to be smoothly varied over a wide range by rotating the magnetization direction in the single F-layer. We have studied the amplitude of the spin valve effect and found the optimal ranges of parameters.

## 1. Introduction

The roadmap for modern superconducting electronics [1] classifies the development and study of superconducting spin switches (valves) as one of the promising directions of development. Such devices are necessary for the construction of cryogenic memory elements [2,3,4,5,6,7,8,9,10], neuromorphic processors [11,12,13,14,15,16], and quantum computers [17,18,19] (including those based on the use of quantum optics effects).

Research in this direction was initiated by theoretical calculations [20,21,22,23,24]. They showed that the critical current $J_{C}$ of Josephson contacts containing two ferromagnetic (F) films depends on the mutual orientation of the magnetization vectors **M**${}_{1,2}$ in these films. Further development of this direction (see reviews [25,26,27,28,29,30]) showed that the presence of two or more ferromagnetic layers in the weak-link region indeed allows for controlling the critical current $J_{C}$ of these junctions by changing the mutual orientation of the magnetization vectors in the films [31,32,33,34]. However, a large number of ferromagnetic layers in the weak-link region is accompanied by degradation of both $J_{C}$ and the characteristic voltage $V_{C}$ of such devices due to a larger number of interfaces in the structure, strong suppression of superconducting correlations in each of the F-layers, and the need to fix the vector **M**${}_{1}$ when changing the direction of **M**${}_{2}$.

In [35,36,37,38,39,40], it has been demonstrated that superconducting spin valves can also be realized with structures containing a single ferromagnetic layer by controlling the position of the maximum in the $J_{C}\left(B\right)$ dependence on the external magnetic field intensity *B*. In magnetic spin valves [41,42], switching of $J_{C}$ was proposed to be achieved by changing the direction of the magnetization vector **M** in the F film’s plane. However, as in the first and second solutions, continuous maintenance of either the magnitude or direction of *B* is required for device operation.

In order to overcome this limitation, the use of SIs-F/N-S contacts was proposed [4], where a thin s-film is in contact with a layer consisting of adjacent end-to-end ferromagnetic (F) and normal (N) regions, and separated from the massive superconducting electrode S by an insulating layer I. Here s-film can be divided into superconducting domains with phases of the order parameters shifted by π relative to each other. However, the practical realization of such a device is a rather complex technological challenge.

A more advanced implementation of Josephson memory with electrical control is based on the coexistence of two metastable states in the area of parameters near the phase transition from the 0- to the π-state [43,44,45,46]. The conditions for such coexistence depends on both the material properties and geometry of the contacts. In SIsFS structures [45,46,47,48], the device can be in either the ground (0) or metastable (π) states, which have similar critical current values. The energy barrier separating these states prevents transitions during a continuous change in the phase difference φ of the order parameters of the S- and s-electrodes. This element stores information only in the phase difference at the contact in a steady state (either 0 or π), and transition between states is achieved by applying a current pulse without magnetization reversal of the F-layers. However, achieving the necessary precision in layer thickness required for memory chip fabrication is a great challenge for this approach.

The next promising direction in the development of superconducting spintronics is the use in spin valves of heavy metals or other materials in which spin–orbit interaction takes place either in themselves or at their boundaries with a superconductor or ferromagnet (see the review [49] and the references therein). In these devices, the critical temperature of the S-layer can be turned either by converting s-wave singlets into other types of correlations, among them s-wave odd-frequency pairs robust to impurity scattering [50,51], or by manipulating the direction or magnitude of the ferromagnetic moment acting on the S-layer [52,53,54,55,56,57,58,59,60,61,62]. The implementation of SOI in the structures reveals the novel class of the spin valve devices that contain the only ferromagnetic layer.

Following this new direction, in this work we propose a design for a Josephson spin valve that allows switching between two predetermined states as well as smooth and large changes in its critical current value. To achieve this, we have essentially used the SN${}_{SO}$F device as one of the electrodes of our device. We have previously shown [62] that the coexistence of Rashba and Dresselhaus SOI in an N${}_{SO}$ film leads to the formation of a significant spin–orbit scattering anisotropy in it. We will show below that it is this anisotropy that opens the way to the realization of a device in which it is possible to realize a smooth variation of the values of its critical current as a function of the direction of the magnetization vector of the F-layer.

The proposed device is a tunnel Josephson contact between a massive superconductor S and a multilayered sN${}_{SO}$F structure consisting of a superconducting film (s), a layer of normal metal (N${}_{SO}$) with spin–orbit interaction, and a monodomain ferromagnetic film (F). It is assumed that the S and s materials are superconductors with conventional S-wave pairing potential; in the N${}_{SO}$ layer, two types of spin–orbit scattering (Rashba and Dresselhaus types) coexist [50,51,59,60]; the direction of the magnetization vector of the upper F layer lies in the plane 0xy and can form an arbitrary angle with the direction 0x.

## 2. Model of the SIsN_*SO*_F Spin Valve

**Figure 1:**
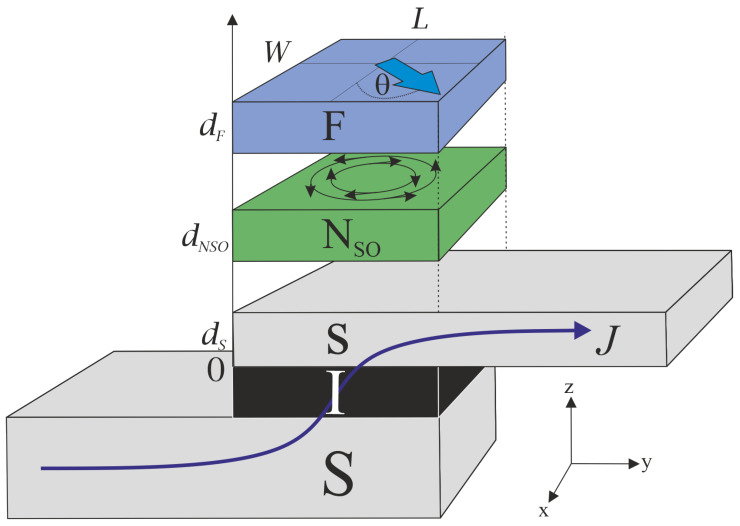
Schematic representation of the considered SIsN${}_{SO}$F spin valve.

The SIsN${}_{SO}$F device under analysis is depicted in Figure 1. It consists of two conventional superconductors (S; s) with singlet pairing potential separated by an insulating layer (I). The upper thin s-electrode is in contact with a bilayer structure composed of a ferromagnet (F) and a normal metal (N${}_{SO}$) with spin–orbit interaction (SOI) of electrons. We will assume that the width of the contact, W, and its length, *L* (in the direction of *y*), are much smaller than the Josephson penetration depth. At the same time, we assume that *L* is much larger than superconducting coherence length, $\xi_{s}$. The latter condition makes it possible to neglect the proximity effect with the part of the upper s electrode protruding from the SIsN${}_{SO}$F structure.

In the ferromagnetic film, spins of the quasi-particles are polarized along the direction determined by the magnetization vector **M**. The isotropic spin–orbit scattering partially destroys [63] this ordering in the N${}_{SO}$-layer. Therefore, the suppression of superconductivity in the SIsN${}_{SO}$F structure with a regular normal metal should be stronger than in the SIsN${}_{SO}$F contact under otherwise equal conditions. However, if the vector **M** lies in the plane of the F-layer and the SOI is anisotropic in the same plane in the N${}_{SO}$ film, then the critical current of the spin valve in the SIsN${}_{SO}$F structure should depend on the direction of **M**. It is known [49,64] that such an anisotropy arises when two types of SOI coexist in the N${}_{SO}$ layer: Rashba [65] and Dresselhaus [66].

The aim of this study is to investigate the influence of such anisotropy on the critical current density $J_{C}$ for SIsN${}_{SO}$F spin valves. The pursuit of this goal is divided into two stages. In the first stage, we will solve the proximity problem in the considered structure and find the values of anomalous Green’s functions at the Is-interface, neglecting the current flowing through it (i.e., assuming its density to be much lower than the depairing current density for the s-film). In the final stage, using the values of these Green’s functions, we will calculate the current through the SIsN${}_{SO}$F-contact as a function of the direction of the vector **M**.

We assume that the “dirty limit” conditions are met for all SIsN${}_{SO}$F materials in the structure, and their material constants (specific resistance ρ, coherence length $\xi ={\left(D/2\pi T_{C}\right)}^{1/2}$, diffusion coefficient *D*, critical temperature of superconductors $T_{C}$) are the same for all metals. The suppression parameters $\gamma_{B}=R_{B}/\rho \xi$ ($R_{B}$ is the specific resistance of the boundary) for sN${}_{SO}$ and N${}_{SO}$F interfaces are also equal to each other.

To be specific, we will also assume that the ferromagnet has a single-domain structure, with its magnetization vector $\vec{M}$ lying in the 0xy plane, and the exchange interaction vector, $\vec{h}$, is as follows:

$$\vec{h}=h\vec{n_{x}}\cos\theta +h\vec{n_{y}}\sin\theta .$$

Here, *h* is the exchange energy, θ is the angle between the 0x axis and the direction of the magnetization vector, $n_{x}$ and $n_{y}$ are unit vectors along the 0x and 0y axes (see Figure 1).

The spin–orbit interaction in N${}_{SO}$ metal is described by the vector $\vec{A}$, which also lies in the 0xy plane.

(1) $$\vec{A}=A_{x}\vec{n_{x}}+A_{y}\vec{n_{y}}=\left(\beta \sigma_{x}-\alpha \sigma_{y}\right)\vec{n_{x}}+\left(\alpha \sigma_{x}-\beta \sigma_{y}\right)\vec{n_{y}}.$$

Here, α and β are the Rashba [65] and Dresselhaus [66] SOI coefficients, respectively, and $\sigma_{x}$ and $\sigma_{y}$ are the Pauli matrices that reflect the structure of the components of the vector $\vec{A}$ in the spin space.

For the given configuration of vectors $\vec{h}$ and $\vec{A}$ the normal, $g_{i}$, and anomalous, $f_{i}$, Green’s functions, which describe the proximity effect in the investigated SN${}_{SO}$F structure, depend only on the coordinate *z* and obey one-dimensional Usadel equations [52,53,54,67].

In the F-film (under the condition $d_{S}+d_{NSO}\leq z\leq d_{S}+d_{NSO}+d_{F}$), the singlet $f_{0}$ and triplet $f_{1,2,3}$ anomalous Green’s functions satisfy the following equations:

(2) $$\begin{array}{c} \begin{array}{c} Df_{0}-i\left(f_{1}h\cos\theta +f_{2}h\sin\theta \right)=0,\\ Df_{1}-if_{0}h\cos\theta =0,\\ Df_{2}-if_{0}h\sin\theta =0,\\ Df_{3}=0; \end{array} \end{array}$$

In the N-layer $\left(d_{S}\leq z\leq d_{S}+d_{NSO}\right)$, the Usadel equations can be written as:

(3) $$\begin{array}{c} \begin{array}{c} Df_{0}=0,\\ Df_{1}-2gD\left(2\alpha \beta f_{2}+\left(\alpha^{2}+\beta^{2}\right)f_{1}\right)=0,\\ Df_{2}-2gD\left(2\alpha \beta f_{1}+\left(\alpha^{2}+\beta^{2}\right)f_{2}\right)=0,\\ Df_{3}-4gD\left(\alpha^{2}+\beta^{2}\right)f_{3}=0. \end{array} \end{array}$$

Finally, in the S-layer $\left(0\leq z\leq d_{S}\right)$, the vectors $\vec{A}$ and $\vec{h}$ are equal to zero, and the Usadel equations are reduced to:

(4) $$\begin{array}{c} \begin{array}{c} Df_{0}+\Delta g=0,\\ Df_{i}=0,\;i=1,2,3, \end{array} \end{array}$$

(5) $$\Delta \left(\ln\frac{T}{T_{c}}+2\pi T\sum_{\omega >0}^{\infty }\frac{1}{\omega }\right)=-2\pi T\sum_{\omega >0}^{\infty }f_{0},$$

where Δ is the order parameter in the s-layer, ω=πT(2n+1) are the Matsubara frequencies (*n* is integer), *T* is the temperature, and the differential operator:

(6) $$Df_{i}=\frac{D}{2}g\frac{d^{2}f_{i}}{dz^{2}}-\frac{D}{2}f_{i}\frac{d^{2}g}{dz^{2}}-\omega f_{i},$$

allows for writing equations in a compact form. The normal and anomalous Green’s functions in each layer are related by a normalization condition, which can be represented in the absence of current flowing through the structure as:

(7) $$g_{i}=\sqrt{1-|f_{0}{|}^{2}+\sum_{i=1}^{3}{\left|f_{i}\right|}^{2}}.$$

Boundary conditions must be added to the system of Equations (2)–(7). At the free surfaces (z=0; $z=d_{S}+d_{NSO}+d_{F}$), we have:

(8) $$\frac{d}{dz}f_{i}=0,\;i=0,1,2,3$$

The aforementioned simple equations are derived from the condition of zero current flow across these boundaries.

At the SN${}_{SO}$$\left(z=d_{S}\right)$ and N${}_{SO}$F $\left(z=d_{S}+d_{NSO}\right)$ boundaries, the Green’s function satisfies the Kupriyanov–Lukichev conditions [68], which are valid at non-magnetically active interfaces [69,70].

(9) $$\gamma_{B}\left(g_{l}\frac{d}{dz}f_{l}-f_{l}\frac{d}{dz}g_{l}\right)=g_{r}f_{l}-f_{r}g_{l}.$$

They are valid for all layer indices, i=0,1,2,3, and relate the functions $f_{i}$ and $g_{i}$ on the left (index *l*) and right (index *r*) sides of each boundary.

To solve the boundary value problem (4)–(9), we have created a software package that can calculate the spatial distributions of Δ(z) and $f_{i}\left(z\right)$ (i=0,1,2,3) within the multilayer SIsN${}_{SO}$F structure for different geometric and material parameters of its layers. The key results obtained are described in the next section.

## 3. Proximity Effect in sN_*SO*_F Structure

An analysis of the SOI influence on proximity effect in sN${}_{SO}$F structure established in [62] demonstrates that the pair potential of the s-layer Δ depends on the angle θ of the F-layer magnetization. At the angle θ=π/4 SOI effectively destroys triplet correlations appearing in the F-layer and protects superconducting order from poisoning. However, at the angle θ=3π/4 SOI ignores triplet correlations and the pair potential of the s-layer is effectively suppressed due to inverse proximity effect.

For further consideration of the devices, we examine the natural parameter $\delta \Delta =\Delta_{s}\left(\theta =\pi /4\right)-\Delta_{s}\left(\theta =3\pi /4\right)$, which corresponds to the difference between the pair potential in the “open” and “closed” states. In other words, the parameter δ is the “strength” of the spin valve effect.

**Figure 2:**
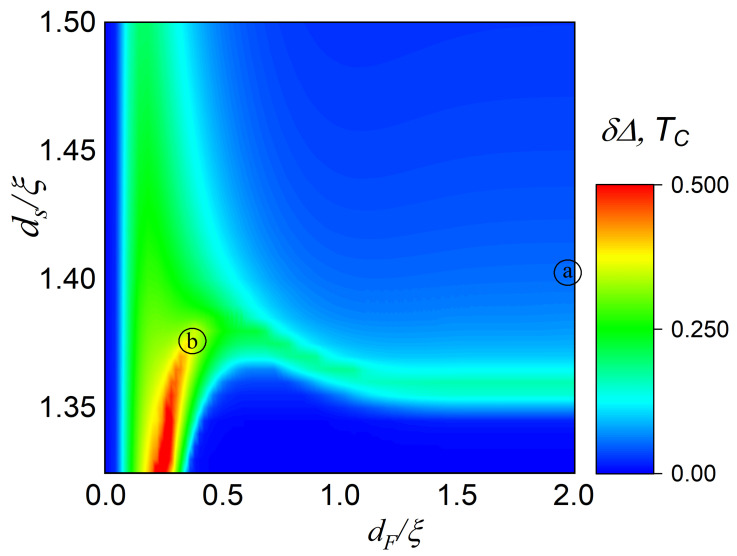
The map of the difference between the “open” and “closed” states, i.e., the order parameters difference $\delta \Delta =\Delta_{s}\left(\theta =\pi /4\right)-\Delta_{s}\left(\theta =3\pi /4\right)$ as a function of s-thickness and F-thickness. The order parameter $\Delta_{s}$ calculated at the Is-boundary by s-layer. The letters in the circle correspond to the thickness sets for Figure 3a and b, respectively. The calculations were made for $d_{NSO}=0.2\xi$, α=β=1, $h=20T_{C}$, $T=0.5T_{C}$, $\gamma_{B}=0.3$.

Figure 2 shows the dependence of δ on the geometric scales $d_{s}$ and $d_{F}$ of the sN${}_{SO}$F structure. The other parameters are typical for hybrid structures: $d_{NSO}=0.2\xi$, α=β=1, $h=20T_{C}$, $T=0.5T_{C}$, $\gamma_{B}=0.3$.

In the case of the thick F-layer $d_{F}>\xi$, a significant spin valve effect appears only in the narrow vicinity of critical thickness $d_{sC}\approx 2.72\xi$. At this thickness the superconductivity in the s-layer is almost suppressed by the proximity effect.

At the same time, in the limit of the thin F-layer $d_{F}\approx 0.2\xi$ the spin valve effect occurs in the wide range of the s-layer thickness. Furthermore, the critical thickness $d_{sC}$ decreases in this case, and the region with the peak spin valve effect appears at $d_{F}\approx 0.2\xi$ and $d_{s}<2.75\xi$.

Finally, at the limit of small $d_{F}\ll 0.1\xi$, the F-layer becomes too small to have any influence, and the spin valve effect disappears in the whole interval of $d_{s}$ thicknesses.

In the following part of the paper we consider two points shown in Figure 2. The first of them (a) corresponds to the structure with thick F-layer $d_{F}=2\xi$ and s-layer in the reasonable vicinity of critical thickness $d_{s}=2.8\xi >d_{sC}$. The second point (b) relates to the case of thin F-layer dF=0.38ξ and s-layer $d_{s}=2.75\xi$, which provides maximal spin valve effect.

We start here with a discussion of the spatial distribution of superconducting correlations in the controllable Josephson device under consideration and its key components.

**Figure 3:**
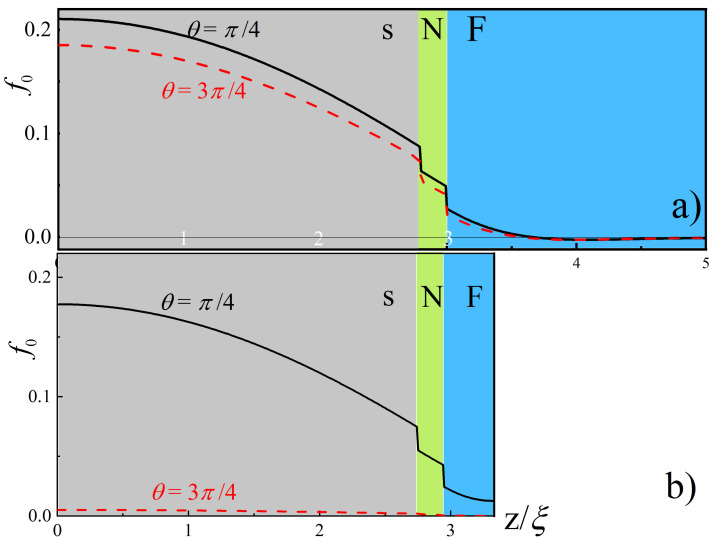
Spatial distributions of the anomalous Green’s function $f_{0}\left(z\right)$ calculated for ω=πT, θ=π/4,θ=3π/4, α=β=1, $d_{NSO}=0.2\xi$, $h=20T_{C}$, $T=0.5T_{C}$, $\gamma_{B}=0.3$. The calculations were made for (**a**) $d_{S}=2.8\xi$, $d_{F}=2\xi$ and (**b**) $d_{S}=2.75\xi$, $d_{F}=0.38\xi$.

Figure 3 demonstrates spatial distributions of the function $f_{0}\left(z\right)$ in the mentioned points (a) and (b). The solid black curves represent the dependence of $f_{0}\left(z\right)$ for θ=π/4, while the dashed red curves show the dependence calculated for θ=3π/4. It can be seen from Figure 3a that for F-layer thicknesses greater than ξ and z≳3.5ξ, the functions $f_{0}\left(z\right)$ calculated for both angles θ coincide and change sign from positive to negative due to the boundary condition (8). As one approaches the N${}_{SO}$F-interface, the difference between the dashed and solid curves increases. This is because at θ=π/4, the presence of spin–orbit electron scattering in the N${}_{SO}$-material leads to an effective reduction in the ferromagnetic ordering of quasiparticles near the N${}_{SO}$F boundary. At the same time, at θ=3π/4, the N${}_{SO}$ layer behaves like a normal metal, so the magnitude of the Zeeman splitting of subbands in the ferromagnet is not modified. This circumstance leads to a stronger suppression of the value of $f_{0}$ at the N${}_{SO}$F-boundary at θ=3π/4. As one moves towards the Is-interface, the difference between the values of $f_{0}$ calculated for different angles gradually increases. Together with this difference, the possibilities for controlling the critical current of the element also increase. The revealed behavior is dictated by the boundary condition (8) at the Is-interface.

The situation changes drastically for small thicknesses of the F-film (see Figure 3b). Earlier this was designated as a case of a strong spin valve effect. At an angle of θ=π/4, the effective exchange energy in the F-layer is significantly suppressed by the transboundary influence of spin–orbit scattering in the N${}_{SO}$-material. This allows for satisfying the boundary condition (8) at the free boundary of the ferromagnet for finite values of $f_{0}$ in its vicinity.

At an angle of θ=3π/4, such suppression of triplet Green functions is absent. As a result, superconducting order is not protected from triplet poisoning, and S-layer is going to the normal state with $f_{0}=0$.

The substantial variation of $f_{0}\left(0\right)$ values at the Is-interface with respect to the angle θ undoubtedly affects the critical current magnitude of the SIs tunnel contact with N${}_{SO}$ controlling element.

## 4. Critical Current of the SIsN_*SO*_F Spin Valve

The critical current magnitude of the SIsN${}_{SO}$F spin valve can be determined using the Ambegaokar–Baratoff formula [71] for asymmetric SIs tunnel contacts:

(10) $$\frac{eJ_{C}R_{N}}{2\pi T_{C}}=\frac{T}{T_{C}}\sum_{\omega }\frac{\Delta_{1}f_{0}}{\sqrt{\left(\omega^{2}+\Delta_{1}^{2}\right)}},$$

where $\Delta_{1}$ is the order parameter modulus in the S-electrode and $f_{0}$ are the values of anomalous (ω-dependent) Green’s functions at the Is-interface (z=0).

**Figure 4:**
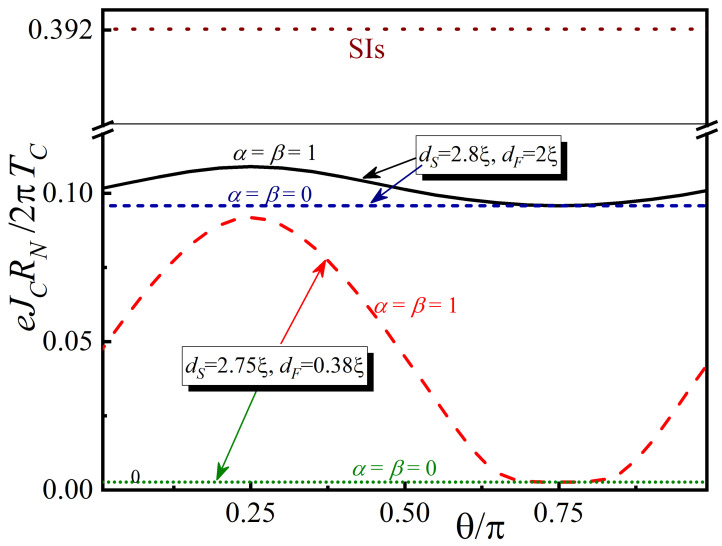
Critical current dependencies for the SIsN${}_{SO}$F spin valve versus the angle θ between the h and n${}_{x}$ directions. The calculations were made for ω=πT, θ=π/4,θ=3π/4, α=β=1, $h=20T_{C}$, $T=0.5T_{C}$, $\gamma_{B}=0.3$, $d_{NSO}=0.2\xi$, and two sets of thicknesses: $d_{S}=2.8\xi$; $d_{F}=2\xi$ and $d_{S}=2.75\xi$; $d_{F}=0.38\xi$. The value of the tunneling critical current between two bulk superconductors is denoted by “SIs”-mark.

Figure 4 shows the dependencies for critical current density, $J_{c}\left(\theta \right)$, calculated at $T=0.5T_{C}$, α=β=1, $h=20T_{C}$, $\gamma_{B}=0.3$$d_{NSO}=0.2\xi$, and two combinations of S- and F-film thicknesses: $d_{F}=2\xi$, $d_{S}=2.8\xi$; $d_{F}=0.38\xi$, $d_{S}=2.75\xi$. The behavior of $J_{c}$ for SIs, SIsNF (Figure 3a), and SIsNF (Figure 3b) tunnel junctions are indicated by brown, blue, and green dashed lines, respectively. “N” here denotes N${}_{SO}$-layer with “turned off” spin–orbit interaction. It can be seen that the proximity effect in the upper electrode of tunnel structures leads to the suppression of the critical current magnitude. For the selected calculation parameters, the minimum suppressed value of $J_{c}$ differs by approximately 8 times from the case of SIs structure. A non-monotonic dependence of $J_{c}\left(\theta \right)$ is observed at $d_{S}=2.8\xi$; $d_{F}=0.38\xi$, in which the difference between the maximum and minimum values of $J_{c}$ is small. However, at $d_{F}=0.38\xi$, $d_{S}=2.75\xi$, this difference is already significant, allowing for smooth and wide-range control of critical current values by changing the direction of the magnetization vector in the F-layer.

**Figure 5:**
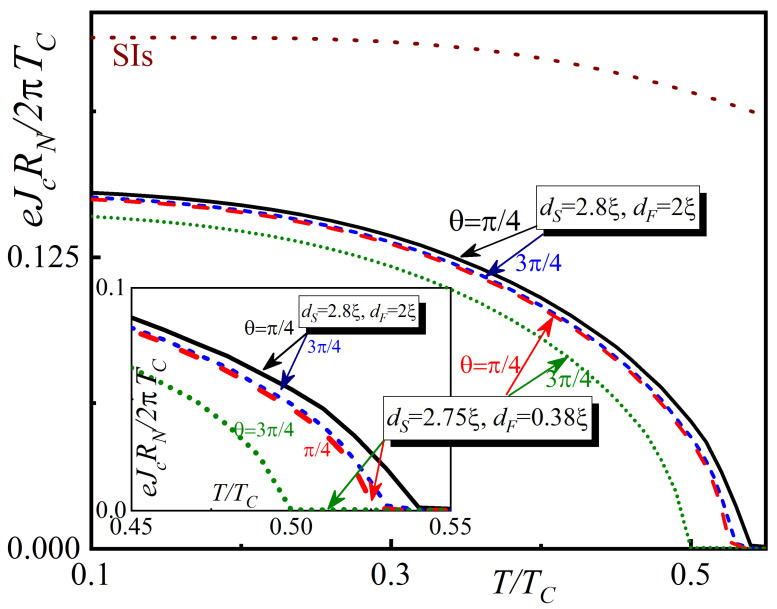
Critical current dependencies for the SIsN${}_{SO}$F spin valve versus the temperature *T*. The calculations were made for θ=π/4 and θ=3π/4, for two sets of thicknesses: $d_{S}=2.8\xi$; $d_{F}=2\xi$ and $d_{S}=2.75\xi$; $d_{F}=0.38\xi$. For every curve α=β=1, $h=20T_{C}$, $T=0.5T_{C}$, $\gamma_{B}=0.3$, $d_{NSO}=0.2\xi$. The value of the tunneling critical current between two bulk superconductors is denoted by “SIs”-mark.

The temperature dependence of the critical current density $J_{c}\left(\theta \right)$ is shown in Figure 5 for the spin valve in the “open” (θ=π/4) and “closed” (θ=3π/4) states. The results are presented again for two sets of parameters: $d_{S}=2.75\xi$, $d_{F}=0.38\xi$ (dashed red and dotted green lines) and $d_{S}=2.8\xi$; $d_{F}=2\xi$ (solid black and short-dashed blue lines). For comparison, the temperature dependence of $J_{c}\left(\theta \right)$ for the trivial SIs transition is shown as a dark red dotted line. The typical feature of the spin valve device is the suppressed critical temperature: the superconducting order parameter appears in the temperature interval T≈(0.5…$0.55)T_{C}$. This region is enlarged in the inset of Figure 5.

At $d_{s}=2.8\xi$ the superconductor is thick enough. The fact that there is a strong suppression of superconductivity at its boundary with a normal metal has a weak effect on the magnitude of the order parameter at its boundary with the tunnel barrier. Therefore, the difference between the calculations performed for angles θ=π/4 and θ=3π/4 is small. This conclusion follows from the calculation results shown in Figure 4. At $d_{s}=2.75\xi$ and $0.5≲T/T_{c}≲0.55$ the s-layer is close to its critical thickness. The inset in Figure 5 shows that for this set of parameters the difference between the critical currents calculated at θ=π/4 and 3π/4 is large. However, as the temperature decreases, the superconductivity in the s-film begins to recover and the difference in critical currents becomes less pronounced.

Therefore, the spin valve effect occurs at any temperature for the presented structures. The highest amplitude of the difference between the currents in the “open” and “closed” states occurs near the critical temperature of the structure.

## 5. Discussion and Conclusions

In summary, the SIsN${}_{SO}$F spin valve examined in this study can serve as a device that smoothly regulates either the magnitude of the supercurrent flowing through it or the inductance of the circuit in which it is included. It is crucial that these parameters can be smoothly and wide-range adjusted by changing the direction of the magnetization vector in the F-layer. This advantageous feature sets apart the proposed technical solution from previously investigated counterparts that exhibited a step-like change in characteristics when switching between only two stable states corresponding to parallel or antiparallel orientations of the magnetization vectors of the ferromagnetic materials involved in the valves [31,32,33,34,61].

In the device we studied, the problem of reducing the inverse effect of the F-layer on the superconductivity of the upper thin s-electrode of the tunnel Josephson junction was solved by using the *anisotropic* spin–orbit N${}_{SO}$ filter. It either completely opened the effect of the F-layer on the s-film, leading to the suppression of superconductivity in it, or significantly weakened this effect. In our device, the problem of minimizing the direct effect of the F-layer on the s-film was not set and not solved. However, if the task of reducing the exchange energy of the ferromagnetic layer is still important, it can be solved by using a ferromagnetic insulator (FI) as the F-layer. In such an S-N-FI structure, the small thickness N of the film allows both to minimize the suppression of superconductivity in the S-electrode due to the proximity effect and to reduce the value of the effective exchange energy h=U(d/ξ) in the N-layer [72]. Where *U* is the exchange energy of the ferromagnetic insulator and ξ is the decay length of the N-layer (the estimation of *h* is valid for the condition d≪ξ).

It should be noted that the problem of creating a high-quality tunnel layer in the SIsNF devices is practically non-existent. Of course, in the process of its fabrication there is a possibility of formation of defects with the tunneling area (pin holes, localized states in the barrier, mechanical stresses, etc.), which can shunt a barrier by providing some direct coupling between the layers [73]. However, it should be noted that in superconducting electronics, this problem has been largely solved in the so-called three-layer technology for the fabrication of Nb-Al-AlOx-Nb tunneling structures [74]. Currently, this technology is widely used both in research laboratories and in foundries and the fabrication processes have been extensively reported [75,76,77,78,79,80].

The smoothness of the variation of the critical current value as a function of the direction of the magnetization vector of the ferromagnetic layer, which we declare, is possible in the absence of both the crystallographic anisotropy and the anisotropy determined by the geometric shape of the layer. The former can be eliminated in an amorphous ferromagnet. The shape anisotropy can be avoided by using an F-electrode in the form of a thin ferromagnetic (round) disc with in-plane orientation of the magnetic moment. Furthermore, in the absence of crystallographic anisotropy, the use of a cogwheel F-electrode can provide the possibility of realizing a device with a step change in the critical current. The synthesis of a suitable material for the normal layer with anisotropic spin–orbit scattering, as well as an amorphous thin ferromagnetic with in-plain orientation of the magnetic moment, is a good task for materials scientists.

## Data Availability

The data presented in this study are available on request from the corresponding author.
